# Testing accuracy in 2D and 3D geometric morphometric methods for cut mark identification and classification

**DOI:** 10.7717/peerj.5133

**Published:** 2018-07-05

**Authors:** Lloyd A. Courtenay, Miguel Ángel Maté-González, Julia Aramendi, José Yravedra, Diego González-Aguilera, Manuel Domínguez-Rodrigo

**Affiliations:** 1 Área de Prehistoria, Universitat Rovira I Virgili Tarragona, Tarragona, Spain; 2 Institut de Paleoecologia Humana i Evolució Social (IPHES), Tarragona, Spain; 3 Department of Prehistory, Universidad Complutense de Madrid, Madrid, Spain; 4 Department of Cartography and Terrain Engineering, Polytechnic School of Avila, University of Salamanca, Avila, Spain; 5 IDEA (Institute of Evolution in Africa), Madrid, Spain

**Keywords:** Taphonomy, Experimental archaeology, Cut marks, Geometric morphometrics, Microscopy

## Abstract

The analysis of bone surface modifications (BSMs) is a prominent part of paleoanthropological studies, namely taphonomic research. Behavioral interpretations of the fossil record hinge strongly upon correct assessment of BSMs. With the significant impact of microscopic analysis to the study of BSMs, multiple authors have discussed the reliability of these technological improvements for gaining resolution in BSM discrimination. While a certain optimism is present, some important questions are ignored and others overemphasized without appropriate empirical support. This specifically affects the study of cut marks. A diversity of geometric morphometric approaches applied to the study of cut marks have resulted in the coexistence (and competition) of different 2D and 3D methods. The present work builds upon the foundation of experiments presented by Maté-González et al. (2015), Courtenay et al. (2017) and Otárola-Castillo et al. (2018) to contrast for the first time 2D and 3D methods in their resolution of cut mark interpretation and classification. The results presented here show that both approaches are equally valid and that the use of sophisticated 3D methods do not contribute to an improvement in accuracy.

## Introduction

Cut mark analysis has had a long history in archaeology. The first studies regarding these traces date back to the 19th century, with the main objective lying in differentiating whether these marks were part of decorations on portable art or if they were the by-product of other types of activities (namely, butchery) (Lartet, 1860; Peale, 1870; Lartet & Christy, 1875). At the beginning of the 20th century, the first experimental works oriented towards explaining these bone surface modifications (BSMs) found in multiple French Palaeolithic sites came to light (Martin, 1906; Martin, 1907; Martin, 1909; Martin, 1907-10). Research in this area, however, went by unnoticed at least until the second half of the 20th century.

During this period (especially after the 1980s) there have been a considerable growth in the number of cut mark taphonomic analyses. For several decades, emphasis was laid on the analysis and interpretation of cut marks (e.g., Binford, 1981; Domínguez-Rodrigo, 1997; Greenfield, 1999; Greenfield, 2006; Nilssen, 2000; Bello & Soligo, 2008; Bello, Parfitt & Stringer, 2009; De Juana, Galán & Domínguez-Rodrigo, 2010; Bello, 2011; Maté-González et al., 2015; Courtenay et al., 2017). Amongst these studies, experimental replication of butchery processes complemented by ethnoarchaeological work with hunter-gatherers have addressed questions related to cut mark anatomical distribution with respect to to different butchery activities, such as defleshing, dismembering, skinning and carcass part selection (e.g., White, 1952; White, 1953; White, 1954; White, 1955; Binford, 1981; Lupo, 1994; Nilssen, 2000; Galán & Domínguez-Rodrigo, 2013; Wallduck & Bello, 2018). Other studies have analysed cut mark frequencies and their location in order to understand different human behavioural processes related to carcass acquisition and butchery (Domínguez-Rodrigo, 1997; Domínguez Rodrigo, 2002; Capaldo, 1998; Lupo & O’Connell, 2002; Domínguez-Rodrigo & Barba, 2005). Another important perspective was added through the analysis and definition of cut marks themselves, by taking into consideration the possible equifinality (lack of resolution to linking effect to cause) produced by other natural phenomena such as trampling or the distorting effect of diagenesis (Binford, 1981; Shipman, 1981; Behrensmeyer, 1984; Fiorillo, 1984; Behrensmeyer, Gordon & Yanagi, 1986; Olsen, 1988; Olsen & Shipman, 1988; Fisher, 1995; Bello, Parfitt & Stringer, 2009; Domínguez-Rodrigo et al., 2009; De Juana, Galán & Domínguez-Rodrigo, 2010; Bello, 2011; Marín-Monfort, Pesquero & Fernández-Jalvo, 2013; Pineda et al., 2014). Equally important was the differentiation of cut marks produced by different raw materials including lithic, metal, wood, bamboo and shell tools (Walker, 1978; Shipman & Rose, 1983; Olsen, 1988; Greenfield, 1999; Greenfield, 2006; Choi & Driwantoro, 2007; West & Louys, 2007; Bello & Soligo, 2008; Bello, Parfitt & Stringer, 2009; Domínguez-Rodrigo et al., 2009; De Juana, Galán & Domínguez-Rodrigo, 2010; Galán & Domínguez-Rodrigo, 2013). Alongside these developments in analogical frameworks for the interpretation of cut marks, other studies have begun to scrutinize the conditions under which these marks are produced and their variability (Lyman, 1987; Gifford-Gonzalez, 1989; Domínguez-Rodrigo & Yravedra, 2009).

All these different lines of research reflect on the importance of cut mark analysis. Some works embrace modern technologies for cut mark identification and interpretation, with a special reference to microscopic analysis, including SEM (Shipman, 1981; Olsen, 1988; Greenfield, 1999; Greenfield, 2006; Fritz, 1999; Smith & Brickley, 2004; Lewis, 2008), binocular microscopes with high resolution images (Shipman, 1981; Olsen, 1988; Greenfield, 1999; Greenfield, 2006; Smith & Brickley, 2004; Lewis, 2008), digital imaging techniques (Gilbert & Richards, 2000), 3D reconstruction (During & Nilsson, 1991; Bartelink, Wiersema & Demaree, 2001; Kaiser & Katterwe, 2001; Crezzini et al., 2014), 3D digital microscopes (Boschin & Crezzini, 2012; Crezzini et al., 2014), the Alicona 3D Infinite Focus Imaging microscope (Bello & Soligo, 2008; Bello, Parfitt & Stringer, 2009; Bello, 2011; Bonney, 2014) and the laser scanning confocal microscope (Archer & Braun, 2013).

Alongside these techniques, aided by high-resolution microscopy, a number of alternative approaches have become popular over recent years offering promising results in establishing the agency in BSM creation. The most important of these approaches is the use of microphotogrammetry and geometric morphometrics with the use of reflex cameras (Maté-González et al., 2015; Maté-González et al., 2016; Maté-González et al., 2017a; Maté-González et al., 2017b), and the use of a DAVID structured-light scanner SLS-2s in both 2D (Maté-González et al., 2017c) and 3D analysis (Courtenay et al., 2017). These types of methodological approaches have recently been complemented by studies using white-light non-contact confocal profilometers using Digital Surf’s Mountains^®^ software (Pante et al., 2017) as well as full 3D morphometrics aided by Bayesian analysis (Otárola-Castillo et al., 2018). Here “full 3D” is understood as the analysis of BSM features in 3D (i.e., the complete mark with its dimensions and shape) as opposed to the bidimensional analysis of 3D properties of BSMs (i.e., mark section angles or metric properties derived from the 3D reconstruction of the mark). This full 3D method has been argued to be better at BSM identification than any of the other methods listed above, considering how 2D approaches exclude vast portions of bone mark morphologies, while “researchers rely heavily on the ability of profiles or ‘slices’ of marks to represent the more complex whole” (Otárola-Castillo et al., 2018; p. 3). Although (Otárola-Castillo et al. (2018); p. 8) emphatically claim that their method improves the quality of cut mark micro-morphology analysis, experimental evidence to support such a claim is lacking. For such a case, a contrasting hypothesis, preferably comparing methods on the same data set, would have been the best testing scenario. For example, this was recently done when testing which technique (3D digital microscope, laser scanner confocal microscopy or micro-photogrammetry) best captures the original cut mark’s morphology (Maté-González et al., 2017b).

We welcome the addition of 3D morphometric analysis as an additional tool to identify and interpret cut marks. Here our goal is to test whether other approaches, referred to by some authors as 2D because they treat 3D-derived information bidimensionally, possess less resolution in identifying BSM types (i.e., cut- or tooth-marks) than the full 3D approach used by Otárola-Castillo et al. (2018) by using complete 3D reconstruction of marks as the unit of analysis. Those bidimensional approaches also qualify as 3D methods, because they reconstruct BSMs tridimensionally prior to selecting 3D-derived units of analysis (e.g., mark section along different parts of the groove) for bidimensional statistical analysis. We are also interested in testing the potential bias attributed to these 2D analyses, considering the misrepresentation of mark morphology when using sections instead of continuous surfaces, as well as the difficulty and bias of identifying homologous landmarks and semi-landmarks and inaccurate estimates of correct mark classifications. If that were the case, classification errors should be higher in assemblages analysed via 2D methods than in 3D methods. In order to test this hypothesis, we replicated the experiments presented by Otárola-Castillo et al. (2018) and compared the results generated using both 2D and 3D analysis of cut mark morphology through our methodology (Maté-González et al., 2015; Courtenay et al., 2017).

## Materials and Methods

Otárola-Castillo et al. (2018) argue that 3D methods are superior to 2D methods and tested this by using two structurally-different types of cut marks: cuts and slices. Cuts were defined as BSMs made with the knife perpendicular to the bone surface. Slices were defined as BSMs made with the knife adopting an oblique angle with respect to the bone surface. As a result, cuts had a more symmetrical groove section in relation to the horizontal cortical surface than slices. These structural differences were easily differentiated when using 3D methods. Otárola-Castillo et al. (2018), however, did not contrast the accuracy of their method with any of the 2D methods available and, therefore, their assertion that 3D methods were superior to alternative methods remained untested.

In order to provide consistency between the sample used and the confidence in the interpretation of the results, and since hypothesis-testing methods were to be used (such as MANOVA) we initially estimated the size of the experimental sample needed to produce reliable statistical estimates. Given that most experimental tests do not consider the impact of Type II errors (false negatives), we initially tested the adequacy of a theoretical sample for a power of 0.80. This is the standard for reliability of minimizing the error of retaining a false null hypothesis (i.e., false negatives) (Cohen, 1988). We did so using the “pwr” R library (using the pwr.t.test function) (version 3.3.4), in which we estimated that in order to have a powerful sample to discriminate two experimental scenarios, in which one could be 80% sure of identifying differences if these existed, and 95% sure that non-significant differences due to random variability would not be declared as significant, one would need a minimum sample size per group of 59 cases to detect a moderate effect (Cohen’s *d* = 0.52).

**Figure 1:**
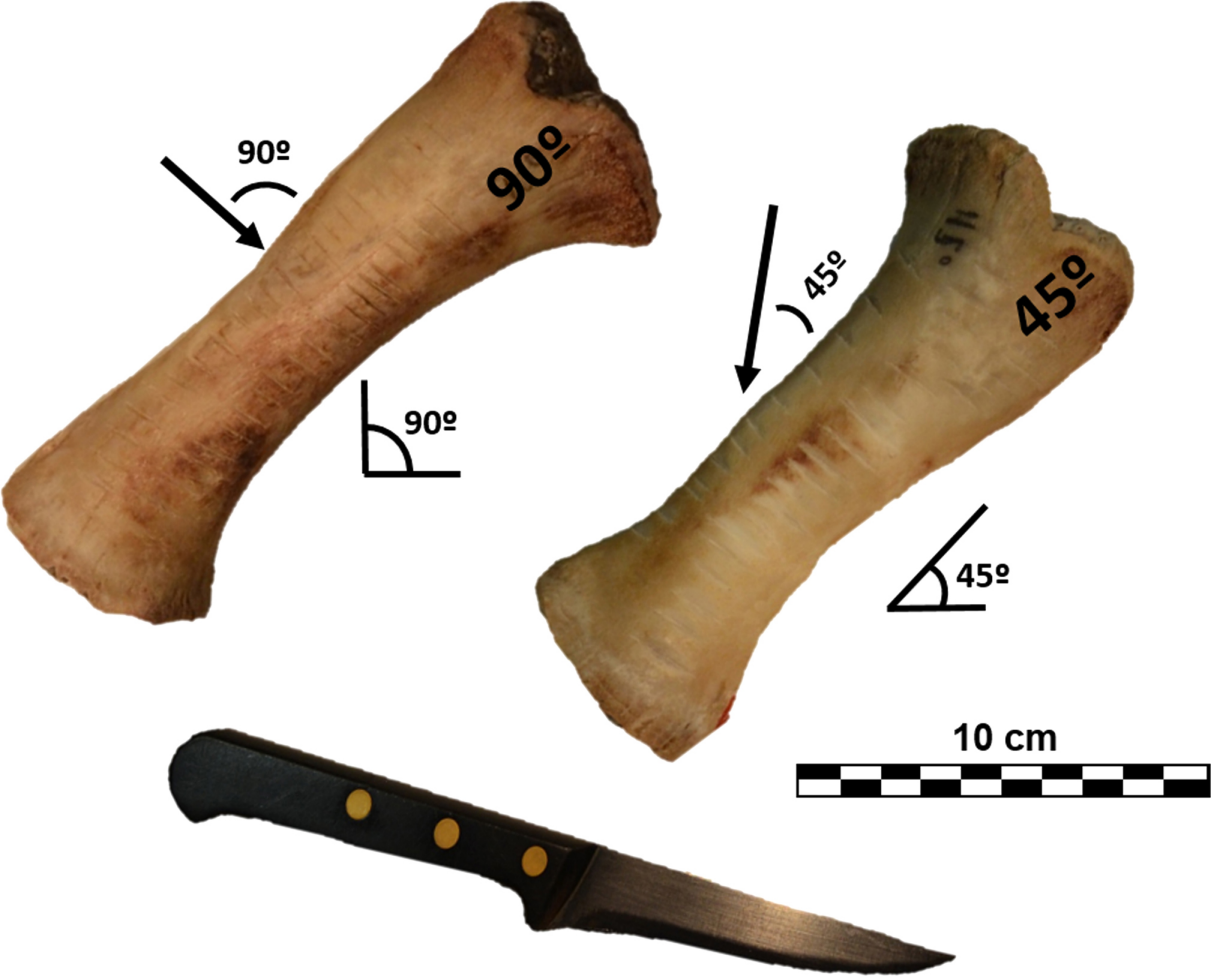
Porcine radii with cut marks performed at different angles along the long axis. Images by Miguel Ángel Maté-González.

Following this a total of 120 cut marks (60 cuts and 60 slices) were generated reproducing the experimental methodology published by Otárola-Castillo et al. (2018) to test mark classification according to cuts (knife perpendicular to bone surface [trend = 90°]) and slices (knife held at acute angle with respect to bone surface [trend = 45°]). The different angles created structurally different cut marks, which could be subsequently tested with 3D and 2D methods. A metal knife model Molybdenum Vanadium C0.5 CR was used to create cut marks on partially defleshed pig bones. During the process the edge was controlled so that no blunting occurred. Half of the marks were inflicted by holding the knife at approximately a 90° angle on 2 radii, and the other half with the tool at approximately a 45° angle along the cranial plane of 3 radii to reproduce cutting and slicing marks, respectively (Fig. 1). All marks were generated with a single motion along the length of the diaphysis, orienting the knife perpendicular to the long axis of the bones. The marks were made by the same butcher who applied similar pressure to all of them. Bones were subsequently cleaned with boiling water and a small solution of neutral detergent.

### Digitalization of marks

The resulting cut marks were digitalized with a DAVID structured-light scanner SLS-2 located at TIDOP at the University of Salamanca (Spain). This laser scanner consists of a DAVID USB CMOS Monochrome camera, an ACER K132 projector, and a calibration marker board. The equipment was calibrated and positioned as explained in Maté-González et al. (2017b).

**Figure 2:**
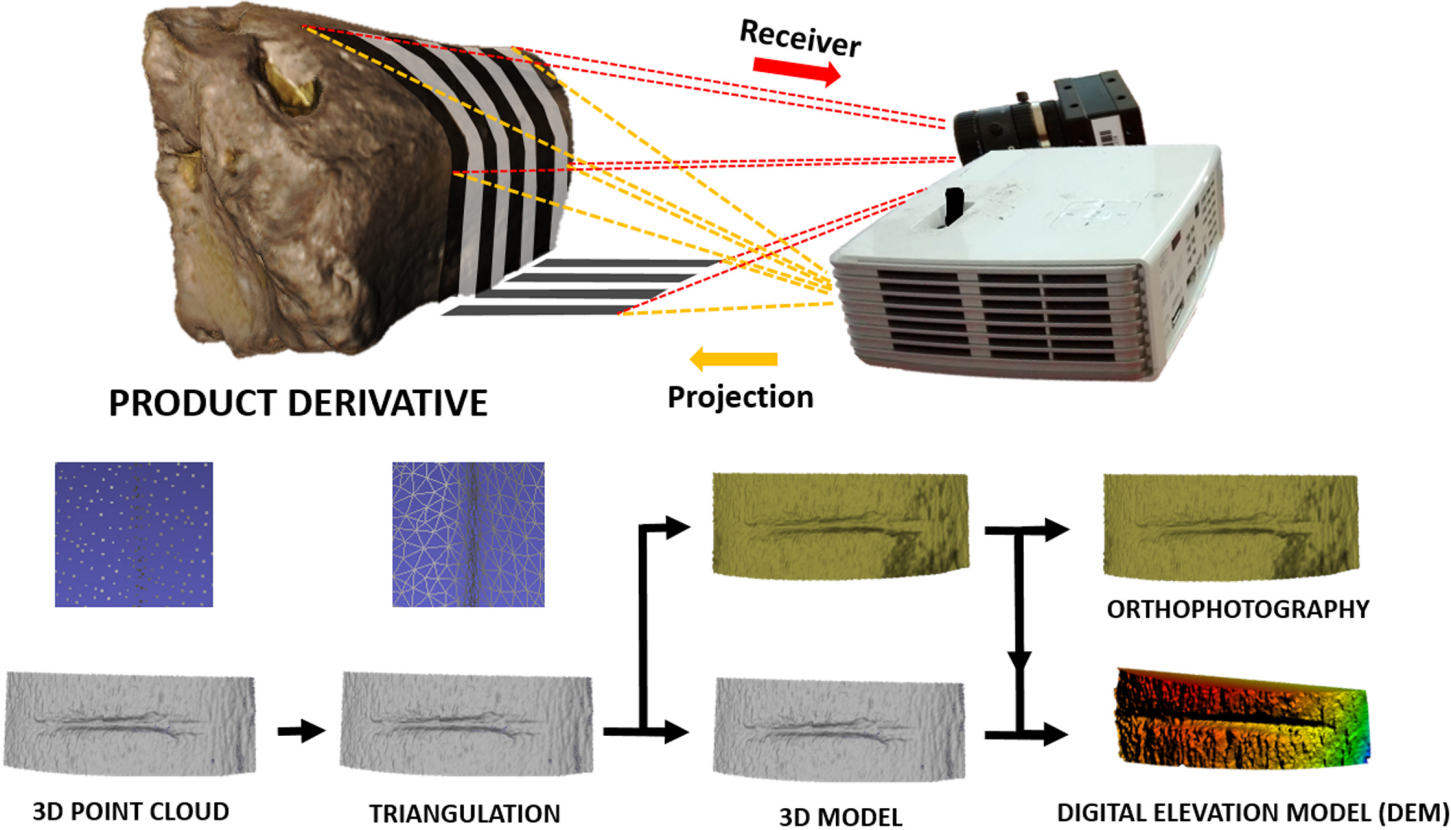
3D scanning using the DAVID structured-light scanner SLS2 and results obtained from data collection. Images by Miguel Ángel Maté-González.

The use of this scanning process provides a 3D surface model of the bone external topography (Fig. 2) in less than 1 min. The DAVID structured-light scanner SLS-2 can produce a density of up to 1.2 million points providing high-resolution 3D models, that can either be directly imported into Avizo (Visualisation Sciences Group, USA) to conduct the 3D analysis (i.e., feature extraction and analysis), or can be treated with Global Mapper software to define mark profiles along the groove. Cut mark sections were obtained at mid-length (always between 30% and 70% of the mark length) as suggested by Maté-González et al. (2015) in order to perform the 2D analysis.

### 2D statistical analysis of cut marks

**Figure 3:**
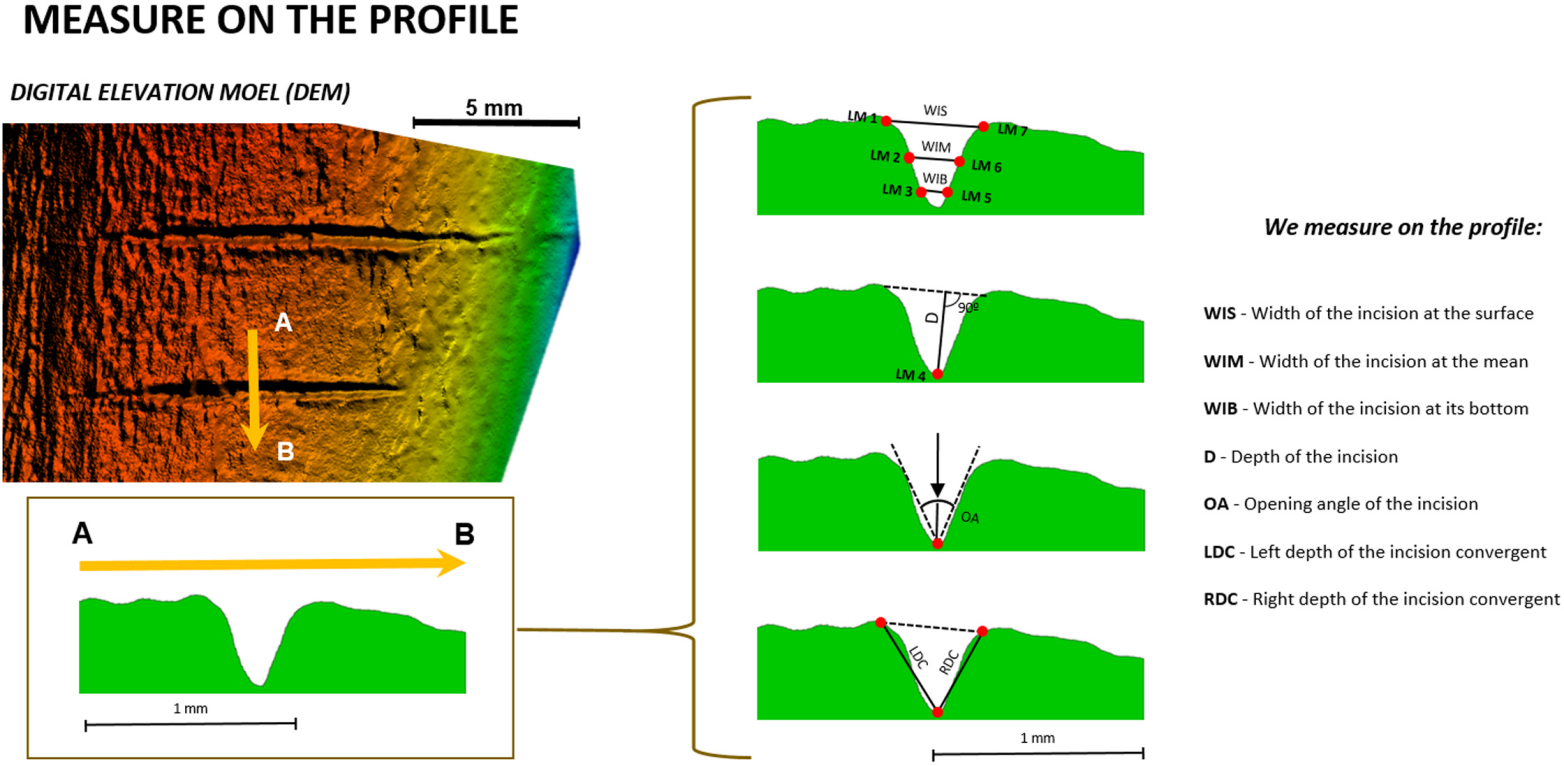
Location of the seven landmarks used in the 2D Morphometric Analysis of cut mark cross-sections (Maté-González et al., 2015) and the measurements taken for each cut mark profile (Bello & Soligo, 2008). Images by Miguel Ángel Maté-González.

First, the free software tpsDig2 (v.2.1.7) was used to take seven measurements (Fig. 3) on the cross-section of each mark (Table 1). Measurements indicating the thickness, depth, and angles of the mark were selected following Bello, De Groote & Delbarre (2013). This biometric data was imported into the free software R (http://www.rproject.org, R Core Team, 2014) to test if cutting (tool at a 90° angle) and slicing (tool at a 45° angle) marks could be differentiated based on simple 2D measurements. If accuracy in the classification of these marks using 2D methods was similar or superior to 3D methods, this would endorse the use of 2D methods in the interpretation of archaeological BSMs.

**Table 1:** Landmarks used in the morphometric 2D and 3D analysis of cut marks.

	No.	Location
**2D model**	1	Beginning of the left line in the mark section
	2	Middle of the left line in the mark section
	3	At 10% of end of the mark on the left line
	4	Deepest point of the mark
	5	At 10% of end of the mark on the right line
	6	Middle of the right line in the mark section
	7	Beginning of the right line in the mark section
**3D model**	1	Beginning of the cut mark
	2	End of the cut mark
	3	Deepest point in the middle of the mark
	4	Left hand shoulder of the middle of the mark
	5	Right hand shoulder of the middle of the mark
	6	Left hand shoulder, halfway between the beginning and the middle of the mark
	7	Right hand shoulder, halfway between the beginning and the middle of the mark
	8	Left hand shoulder, halfway between the middle and the end of the mark
	9	Right hand shoulder, halfway between the middle and the end of the mark
	10	Left hand shoulder, at the opening angle of the mark
	11	Right hand shoulder, at the opening angle of the mark
	12	Left hand shoulder, at the closing angle of the mark
	13	Right hand shoulder, at the closing angle of the mark

Multivariate analysis of variance (MANOVA) was applied to statistically assess the presence of separate groups (cuts and slices) by comparing their means. The MANOVA.RM package (Friedrich, Konietschke & Pauly, 2018) in the R environment was preferred to conduct the analysis after confirming that when using the MVN package (Korkmaz, Goksuluk & Zararsiz, 2014) the condition of variance homogeneity was not fulfilled. The MANOVA.RM package includes variance analyses that do not assume multivariate normality or homogeneity.

The Principal Components Analysis (PCA) included in the FactoMineR library (Lê, Josse & Husson, 2008) was applied to the seven variables described in Table 1 to assess patterns of variation among the data and define the weight of the explanatory variables contained in the sample. PCAs were made using the correlation matrix. In the PCA, each cut mark is a single point, which can be easily plotted in a graph. Plots were made using the ggplot2 R library (Wickham, 2009).

A jackknife cross-validated Linear Discriminant Analysis (LDA) was conducted to determine differences among the two a priori established groups (cuts and slices) by calculating confusion matrices (Efron & Stein, 1981). This method is based on an iterative process that generates random data samples from the population under study by systematically leaving one observation out at a time. Sensitivity was considered adequate given the number of variables and sample size. The LDA function included in the MASS R package was used.

The magnitude of the differences calculated by means of MANOVA and LDA was further tested with an estimation of the effect size using Cohen’s d (Cohen, 1988). Group means and standard deviations were first calculated and the commonly used approach based on dividing the difference between the group means by the pooled standard deviation was applied.

Because one of the variables suggested by Bello, De Groote & Delbarre (2013) had a major impact on the variance explanation, tests were also performed without considering the opening angle of the incision (OA). Additionally, a geometric morphometric analysis was performed. All 2D profiles were landmarked in tpsDig2 (v. 2.1.7) using seven homologous landmarks (Table 1, Fig. 3).

The resulting files containing the 2D landmark coordinates were edited and imported into MorphoJ (Klingenberg, 2011). This software is based on a full Procrustes fit and an orthogonal tangent projection (Dryden & Mardia, 1998) that prepares the sample for usual multivariate statistical analyses. This technique, commonly known as generalized Procrustes analysis (GPA), standardizes the form information by the application of superimposition procedures that involve the translation, rotation, and scaling of the shapes. The remaining differences among the structures under study expose patterns of variation and covariation that can be assessed by means of several statistical tests (Slice, 2001; Rohlf, 1999).

A PCA in shape space, carried out on Procrustes superimposed landmarks, was performed in Morphologika 2.5 (O’Higgins & Jones, 1998) where changes in shape were also visualized with the aid of transformation grids (Bookstein, 1989). PCA scores were later used to examine variance (MANOVA) between the two groups and to estimate the power of discrimination between cutting and slicing marks by means of a jackknife cross-validated LDA. The amount of PC scores used to conduct the MANOVA was limited to gain power performance. For the 2D shape analysis the first 5 PC scores were selected, as they account for almost 99% of the total variance. Before selecting the PC scores, we made sure that no important information was thrown away by observing the correlation of the landmarks with each PC score in MorphoJ and Morphologika 2.5. The LDA test on the shape data were performed using all PC scores calculated by the PCA.

### 3D statistical analysis of cut marks

**Figure 4:**
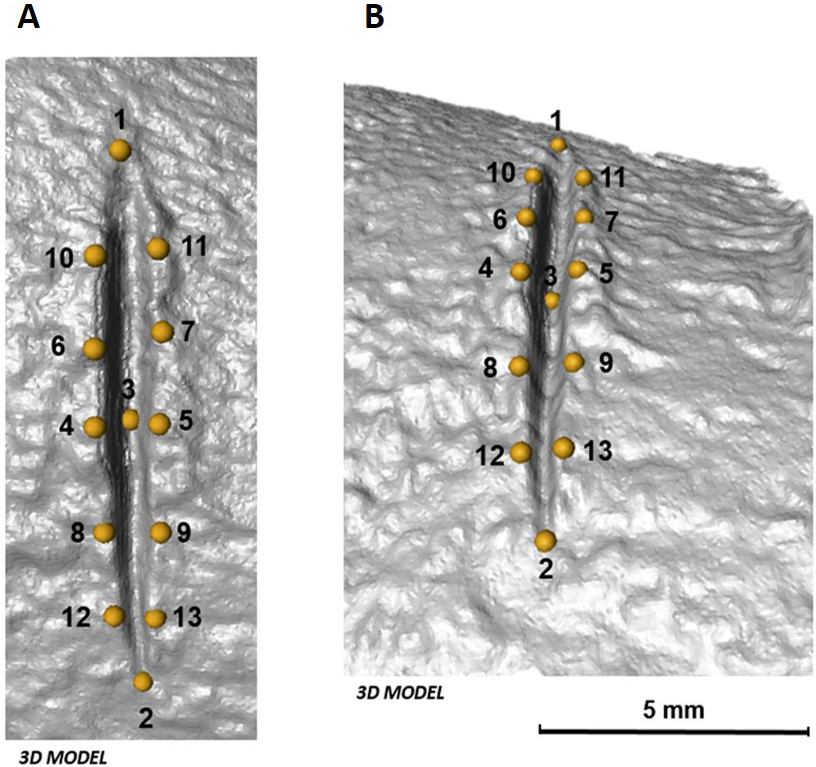
Location of the 13 landmarks used to capture the shape of each mark, as described by Courtenay et al. (2017). (A) Orthogonal view and (B) lateral view of each cut mark in 3D. Images by Miguel Ángel Maté-González.

The 3D landmark configuration consists of 13 identical points on the exterior and interior surface of each cut mark (Table 1, Fig. 4). Following Courtenay et al. (2017), the 13 landmarks that represent qualitative features were established using Avizo (Visualisation Sciences Group, USA) only when their location was unambiguous.

Geometric morphometric analyses were performed in the same manner as in 2D analyses. Additionally, along with morphometric analyses in shape space, form space was investigated after re-scaling the data obtained after Procrustes superimposition using the natural logarithm of Centroid Size. PCAs in shape and form space were computed in Morphologika 2.5 (O’Higgins & Jones, 1998) to assess patterns of variation among the data considering shape and size differences. Changes in shape and form were visualized in the form of transformation grids and warpings computed using thin-plate splines (Bookstein, 1989).

The PC scores obtained in shape and form space were exported into R to examine differences between the two groups of cut marks. In addition, MANOVA and LDA tests were also carried out in R to determine if, on a statistical level, slicing and cutting marks could be distinguished, and to define the classification rates based on the 3D model, respectively. LDA tests were performed using all PC scores calculated by the PCAs in shape and form space, but only the first 10 PC scores were needed to conduct the MANOVA tests as they account for 93.7% of the shape variance and 98% of the total form variance. The magnitude of the differences was tested by calculating the effect size according to Cohen’s d (Cohen, 1988).

## Results

### 2D Analysis

**Figure 5:**
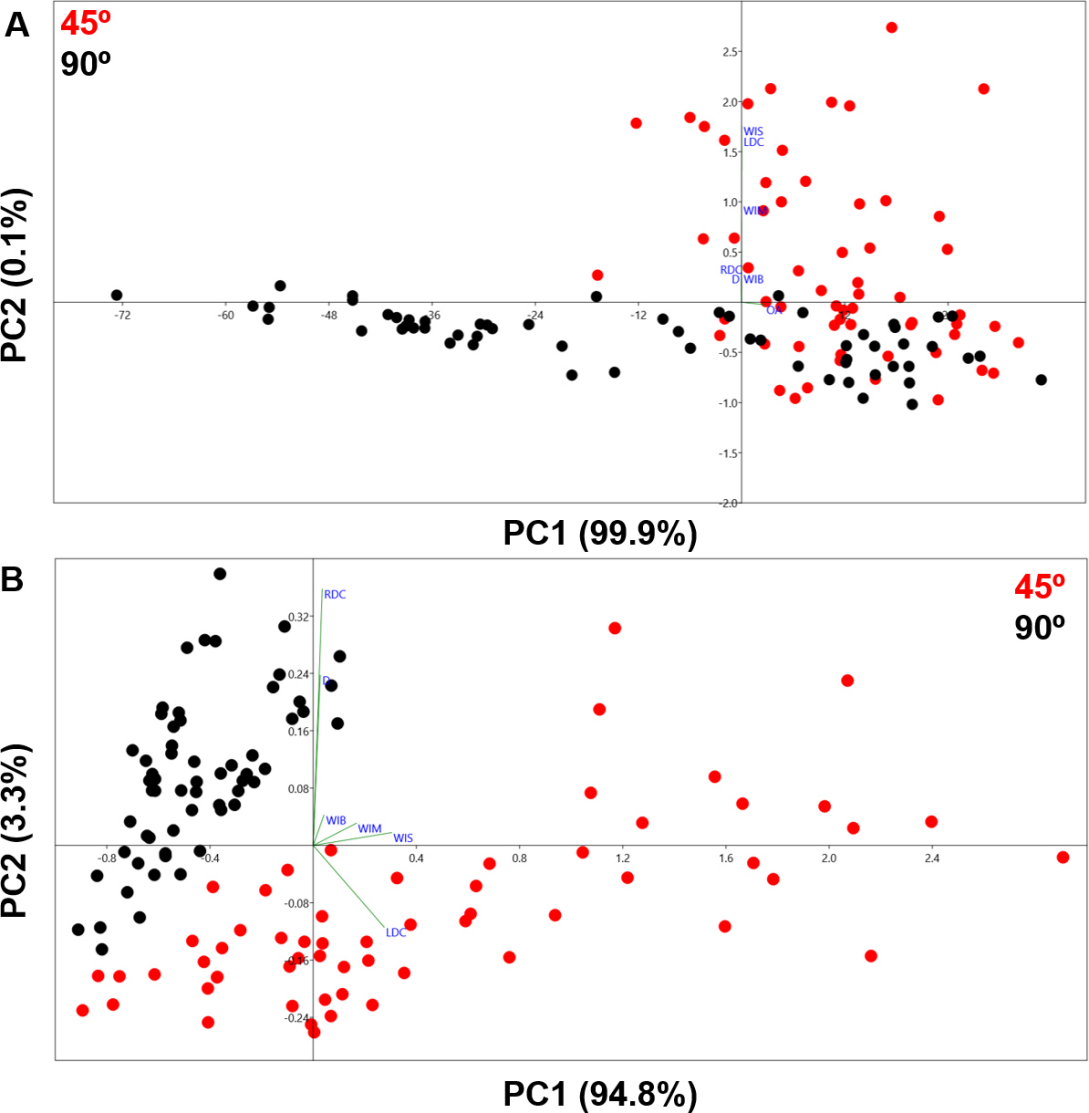
PCA plots in shape space. (A) Including all measurements. (B) Excluding OA.

The PCA analysis of cut marks represented in Fig. 5 present clear differences between the two groups representing different cutting angles. The first two components represent a very high percentage of the sample variance (100% in A and 98.1% in B). The two types of cut marks are differentiated along the first axis in A, which embodies most of the within-sample variance, and a combination of both axes in B. While the exclusion of the opening angle variable in B allows a clearer separation of the two types of cut marks, the inclusion of this variable still enables the differentiation of two clear patterns separating the samples. These results are strongly supported by the numeric results presented in Tables 2 and  3 through significant *p* values in the case of the MANOVA tests and at least 95.83% of the sample being correctly classified in the confusion matrix in the LDA. Though differences between the two groups are very much expressed by the opening angle of the incision as cut marks are inflicted by holding the tool either perpendicular or at acute angle with respect to the bone surface, the Cohen’s d stresses that the magnitude of the differences among groups is greater when the angle measurement is excluded from the analysis (Table 4).

**Table 2:** MANOVA results for the comparison between slicing (45°) and cutting (90°) marks.

	*F*	*p*
All 2D biometric measurements	31.6	<0.0001
2D biometric measurements excluding OA	54.87	<0.0001
2D shape space	138.1	<0.0001
3D shape space	52.09	<0.0001
3D form space	48.53	<0.0001

**Table 3:** LDA confusion matrix results and average % of correctly classified cut marks.

		45°	90°	%correct
All measurements	45°	57	3	
	90°	2	58	95.83%
Measurements excluding OA	45°	58	2	
	90°	1	59	97.5%
2D landmarks in shape space	45°	59	1	
	90°	0	60	99.17%
3D landmarks in shape space	45°	58	2	
	90°	3	57	95.83%
3D landmarks in form space	45°	57	3	
	90°	3	57	95%

**Figure 6:**
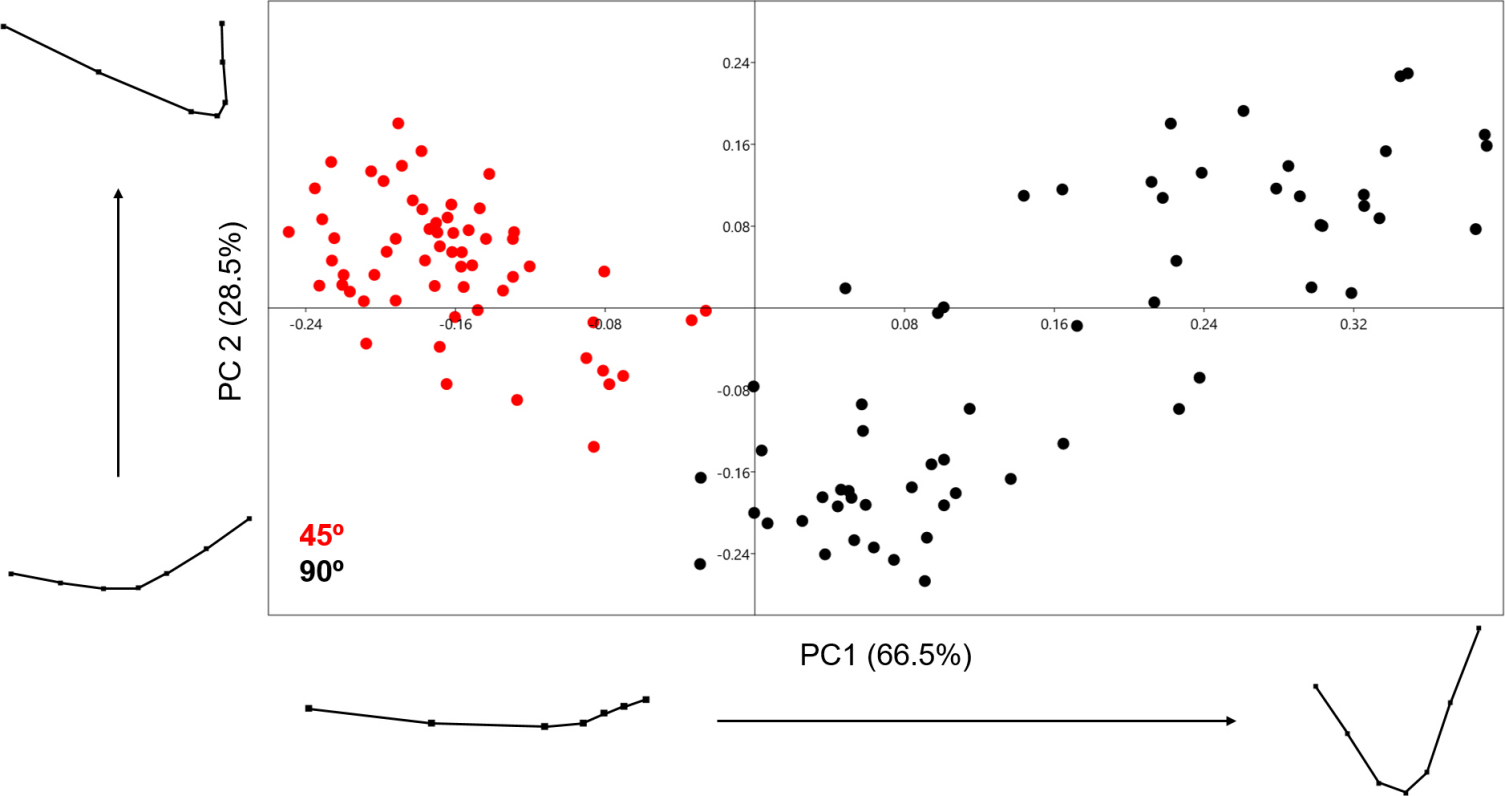
PCA graph presenting variance in cut mark cross-section shape using the 7-landmark model. Variance in shape is presented for the extremities of both PC scores along their respected axis.

The PCA generated using the 2D 7-landmark model yielded a two-component solution that accounts for 95% of the sample variance as shown in Fig. 6. The exceptional distribution data in the Euclidean space shows a complete lack of overlapping samples, clearly separating the two cut mark types in two separate groups. Both groups show no overlap along PC1, which represents changes in cut mark depth and opening angle. Cut marks created with the tool perpendicular to bone surface are narrower and deeper than those created with the tool held at oblique angle. The second PC expresses changes in the opening angle of the mark and the relative proportion of each side. Cutting marks (trend = 90°) show a greater variance pattern along the first two PCs than slicing cut marks (trend = 45°), suggesting that an oblique position of the tool leaves less room for morphological variance. Pairwise MANOVA tests (Table 2) derived from the PC scores differentiate perfectly both samples, producing *p* values of 4.475e−52 (*F* = 138.1). Exploring the variation across the PC morphological scores, we see a great variation in depth as well as the opening angle of the cut mark; represented strongly through changes in all of the landmarks across both axis of the graph (Fig. 6). Classification tables in the LDA matrix are able to correctly classify 98 to 100% of the sample to their correct group (Table 3). The calculated Cohen’s d represents a medium effect size that confirms the existence of differences among groups (Table 4).

**Table 4:** Cohen’s *d* and Effect-size for the comparison between slicing (45° ) and cutting (90° ) marks.

	Cohen’s *d*	Effect-size *r*
All 2D biometric measurements	0.0847	0.0423
2D biometric measurements excluding OA	0.6053	0.2897
2D shape space	−0.4986	−0.2419
3D shape space	0.3172	0.1567
3D form space	0.5375	0.2596

### 3D Analysis

**Figure 7:**
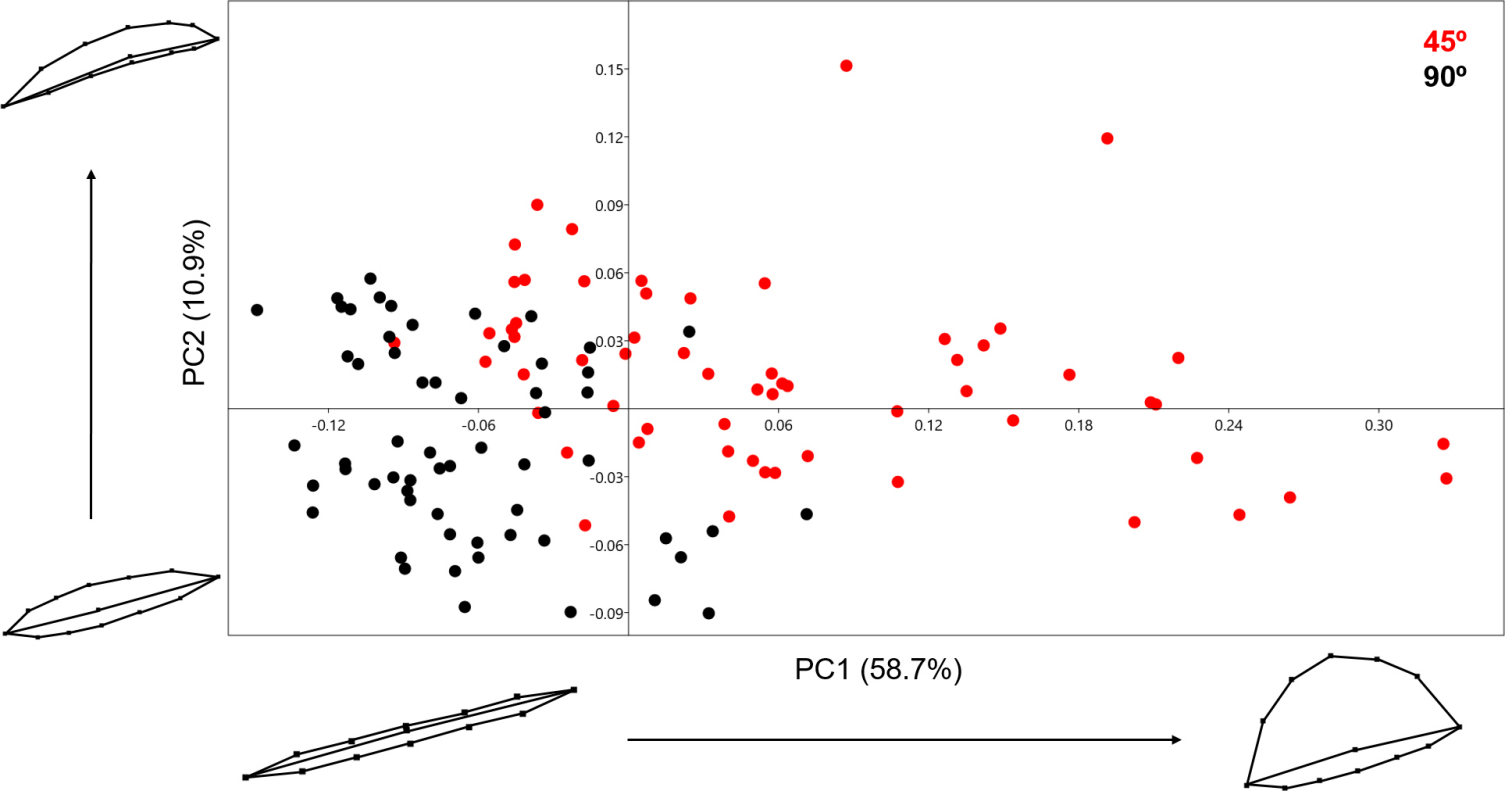
PCA graph presenting variance cut mark shape using the 13 landmark 3D model. Variance in shape is presented for the extremities of both PC scores along their respected axis.

Analysing the samples using the 13-landmark 3D model was able to produce a two-component solution representing a total cumulative variance of 69.574% (Fig. 7). This variance is lower than the variance produced by the 2D PCA. While a certain degree of overlapping can be observed in this graph, taking into consideration the nature of the 13-landmark model, it is understandable that the 3D model is conditioned by more variables than the 2D model. The PCA results, however, are still fairly clear and present two different patterns across the two PC components presented in Fig. 7. Exploring the variation in shape, the majority of variation consists of the positioning of the landmark that marks the middle-lowermost point of the cut mark: highlighting the depth and angle of the mark. PC1, however, also presents a great deal of variability regarding one particular edge of the cut mark, highlighting the angle of the incision. In contrast to the previous PCA scatter plots, here slicing marks (trend = 45°) show a wider dispersion range than cut marks (trend = 90°). When the entirety of the mark is observed, marks produced at an acute angle with respect to bone surface show more morphological variance because the mark is less homogeneous along its length. Cut marks appear to be almost symmetrical and similarly wide along their length. Changes expressed by PC2 are more subtle and do not relate to the longitudinal symmetry as PC1. Cut and slice marks overlap more along PC2, but show opposite trends towards the negative and positive area of the *y*-axis, respectively. The MANOVA results (Table 2) are perfectly capable of differentiating between groups through significant differentiation (*p* = 1.119e−38) of both samples (*F* = 52.09). In this case, the classification/misclassification matrix is able to correctly assign between 95 and 97% of the sample to their correct group (Table 3). However, these differences that tentatively allow the distinction between cut and slice marks are not large according to the Cohen’s d (Table 4).

**Figure 8:**
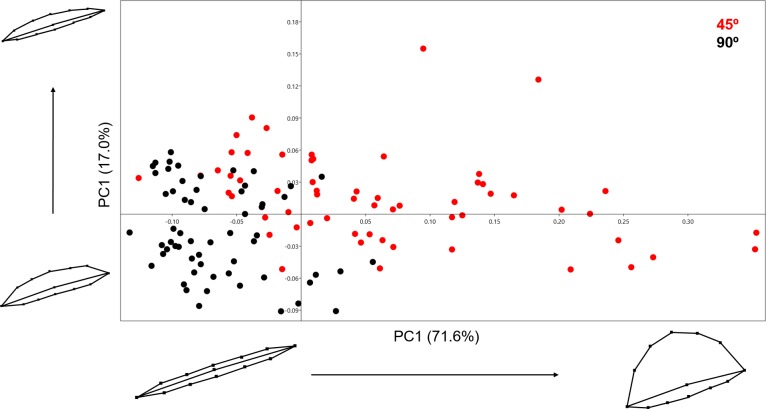
PCA graph presenting variance in cut mark form using the 13 landmark 3D model. Variance in form is presented for the extremities of both PC scores along their respected axis.

Analysing the 13-landmark model including the variable of Procrustes form space produced a similarly successful PCA graph (Fig. 8). The PC scores presented in Fig. 8 represent an even higher portion of the sample variance than when the analysis was carried out excluding form, producing a cumulative variance of 88.5% of the sample (71.6% of which is distributed along the first component). The exploration of form change across the PC scores describe incredibly similar results to the analysis excluding size, highlighting an important variance in the curvature of the cut mark’s walls as well as the angle of the incision. Only subtle differences are observed in comparison to shape changes, with form variance expressed in PC2 being most affected by the inclusion of size. While a certain degree of overlapping is observable through these PCA results, the two samples are still statistically distinguishable through a multivariate analysis (Table 2) with a *p* value of 1.27e−37 (*F* = 48.53). Classification/misclassification tables in this case show that the LDA is capable of correctly distinguishing a total of 95% of the sample (Table 3). Though these results are very similar to the results obtained in shape space, the inclusion of the size variable increases the magnitude of the differences among groups (Table 4).

Regarding the differences between the 2D analysis of cut mark profiles and those resulting from the use of the 13-landmark model to analyse the entire 3D cut mark morphology, these can be logically explained considering the nature of both samples. It would be rational to assume that the angle of incision would greatly affect the angle of the mark; thus impacting all seven landmarks and measurements taken during the analysis of the cut mark profiles. The 13-landmark model explaining the entire morphology of the mark, in fact, analyses the shape, curvature and width of the mark whereas the angle of the incision is represented almost solely through landmark number 3. Thus, we can observe strong variations in the landmarks regarding one edge of the mark in the 3D experimental sample; however, this variation is not as strong in the analysis of mark profiles through 2D. The accuracy of the 2D results is slightly higher than the 3D model; however, both models are still capable of producing highly similar results when splitting and correctly classifying the samples. The significance of these results, as seen through all the statistical tests applied in this paper, provide remarkably clear differentiation between both cut mark samples.

## Discussion

Although Otárola-Castillo et al. (2018) refer to other geometric morphometric approaches (i.e., those used by Maté-González et al., 2015; Maté-González et al., 2016; Maté-González et al., 2017a; Maté-González et al., 2017b) as bidimensional, the truth is that those methods are tridimensional in the way information is derived and sequentially bidimensional (i.e., multidimensional) in the way data are interpreted. Given that these approaches rely on 3D-micromorphology of marks, it would be erroneous to qualify them as bidimensional. Likewise, the presentation of the development of BSM analysis in taphonomy by Otárola-Castillo et al. (2018) is incomplete. For example, Domínguez-Rodrigo & Yravedra (2009) actually argue that regardless of their variability, specific butchery processes are characterized by specific cut mark frequency ranges, which can be used to differentiate certain behaviors. Otárola-Castillo et al. (2018: p. 3) argue that 2D approaches are insufficient because “2D profile data observed on 3D models do not fully exploit the 3D morphological information encapsulated in the BSMs models”. If that were the case, we would observe that 3D morphometric models should yield higher classification accuracy than 2D models. Here, we have shown that this is not the case. Therefore, 2D models seem to capture by a sequence of sections the same shape information as 3D topography. Otherwise said, if 2D methods did not capture enough of the 3D topography of BSMs, that would not be relevant because 2D models show similar (even slightly) higher rates of correct mark classification as 3D models. Otárola-Castillo et al. (2018) likewise complain that these 2D models are affected by irregular use of landmarks. Here we have shown that 2D models use a semi-landmark system by regularly spacing each landmark on the same portion of each mark, as is typical of geometric morphometric analyses in Fourier systems. As proof that this method does not impact the accuracy of 2D methods, it suffices to compare the accuracy of these with that resulting from 3D models. In essence, no argument has been empirically provided to suggest that this is a problem that can impact the method’s heuristics or its “statistical properties, power, bias and error”.

In contrast, this is not something that could be argued in the case of in Otárola-Castillo et al. (2018)’s own analysis. Although they use exploratory methods, Otárola-Castillo et al. (2018) also use hypothesis-testing methods (e.g., MANOVA). The multivariate approach to power analyses of multidimensional designs are more demanding than simple *t*-test methods regarding power. The power in Otárola-Castillo et al. (2018)’s analysis is questionable, since they used a two-sample experiment with 43 marks. In order to differentiate moderate effect sizes between both samples for a simpler *t*-test model, a power analysis shows that they would have needed a much larger sample (Cohen’s delta = 0.5), comprising a minimum of 85 cases per sample (total = 170) if aspiring to a power of 0.9. If lowering the power to 0.8 and aiming at detecting minor differences between both samples, they would have needed a total sample of 175 marks per group. Their current sample size (17 slices and 27 cuts) shows a power of 0.16 to detect small effect sizes (0.3) and of 0.36 to detect moderate effect size (0.5); that is, the probability of having Type II errors in their sample range from 0.84 to 0.64. Although Otárola-Castillo et al. (2018) are not performing comparative metric analyses but classification tests, the small sample they used can also affect the classification rates they derived and the multivariate analysis of their data, especially the MANOVA results (Chartier & Allarie, 2007; Faul et al., in press).

An additional element of concern is that 2D analysis use mark sections that (if the camera is properly calibrated) do not distort the original shape of the section. In contrast, 3D data retrieved through confocal microscope needs a transformation of the raw data for model creation and cleaning that commonly does not reproduce the mark with its exact shape but a distortion thereof. Eventually, the process ends up with a “smoothing” of the surface that “removes any remaining extraneous variation”, including the original variation introduced by the roughness of the mark surface. Additionally, an algorithm is applied, which helps select alignment point in a similar fashion to landmarks in bidimensional studies, which are also non-homologous. This smoothed surface reproduces a proxy of the mark shape that is not the original mark shape. Otherwise put, sections of this smoothed mark may differ from the original shape more than sections of the same mark taken by 2D approaches on the original photogrammetric reconstruction of the mark. The typical angularity of the cut mark is lost through this smoothing process as can be seen in the “taco shell-shape” resulting thereof in Otárola-Castillo et al. (2018)’s Fig. 5. Otárola-Castillo et al. (2018; p. 8) admit additional bias when they acknowledge that “subjective variation could have been introduced during the mark selection and isolation steps. However, if subjective error was added, it was likely introduced to all specimens in a random manner. Consequently, such error is unlikely to have affected the differences between groups”. This remains to be tested. What has not been discarded in the accuracy rates obtained by Otárola-Castillo et al. (2018) is that the two comparative data sets were made on structurally different marks (slices and cuts). The different angle of orientation in the production of these marks created structural differences that the smoothing process did not mask. One set displayed a trend for symmetry and the other set displayed a trend for the contrary. This makes mark distinction fairly easy, even if using hand-lenses and subjective individual assessment. The full 3D method would have benefitted from having tested more complicated scenarios in which BSMs are structurally similar (e.g., trampling marks and cut marks made with retouched flakes). In this more challenging experimental scenario, 2D methods have succeeded repeatedly in differentiating structurally-similar marks (Maté-González et al., 2015; Maté-González et al., 2016; Maté-González et al., 2017a; Maté-González et al., 2017b). It remains to be tested if the full 3D method can pass similar tests with such high accuracy rates as those documented in 2D methods. It would be interesting to see how Otárola-Castillo et al.’s (2018) 3D method works distinguishing morphological BSM variability produced by raw material type (e.g., flint, basalt and quartzite) (Maté-González et al., 2016; Maté-González et al., 2017a; Yravedra et al., 2017a; Yravedra et al., 2017b) or the BSMs differences caused by different tool types (Courtenay et al., 2017). In this regard, Otárola-Castillo et al.’s (2018) method does not present any improvement in the interpretation of cut mark analysis that was not achieved through the use of photogrammetric techniques.

In sum, the present work shows that full 3D analysis of BSMs is a great addition to the range of microscopic and photogrammetric tools available for BSM identification and classification. However, contrary to claims of a higher accuracy yielded by the 3D methods, here we have shown that 2D methods match (and even surpass) classification rates yielded by 3D methods. The rates of both methods in the present work (>95%) are higher than those reported by Otárola-Castillo et al. (2018) for a similar experiment (accuracy = 88%). This difference remains unexplained. The lack of mark surface distortion by smoothing in the 3D method applied in the present work (in contrast with those applied by Otárola-Castillo et al., 2018) may be in part responsible. Since one argument that potentially limits the potential of 3D methods is that they evaluate artificially distorted marks, since they are unable to faithfully reproduce the micro-topography of BSMs and generate their topography through computer algorithms. The higher classification rate obtained here detracts any arguments against the lack of capability of the 2D method in capturing all the essentials of the mark morphology through either sections at the same intervals or semi-landmarks also placed at the same intervals. The results presented here also detract the argument that full 3D methods improve on the heuristics of 2D methods. Both seem to work equally well, and we should all be glad for it.

The claim that Otárola-Castillo et al. (2018)’s work presents the first 3D analysis of BSMs need nuancing. While several papers have been published presenting the use of 3D images in BSMs (Bello & Soligo, 2008; Bello, Parfitt & Stringer, 2009; Bello, 2011; Bonney, 2014; Boschin & Crezzini, 2012; Crezzini et al., 2014; Maté-González et al., 2017b) some authors have even worked directly with these 3D digital reconstructions to create statistical models (Aramendi et al., 2017; Courtenay et al., 2017).

## Conclusions

The response to the use of new 2D–3D techniques in BSM analysis has been mixed. Regarding photogrammetric techniques, contrasting arguments have been made regarding the quality of the results and resolution as opposed to the results produced by the Alicona 3D Infinite Focus Imaging microscope or the laser scanning confocal microscope. These arguments have even been extended to the use of reflex cameras (Maté-González et al., 2017b) or the DAVID structured-light SLS-2s scanner (Courtenay et al., 2017; Maté-González et al., 2017c) which allow the differentiation of not only cut marks but have also been applied to the analysis of BSMs produced by different carnivores (Aramendi et al., 2017; Arriaza et al., 2017; Yravedra et al., 2017c).

Otárola-Castillo et al. (2018) present an experiment comparing the morphological differences of cut marks produced through slicing (45°) and cutting (90°). Through statistical analysis of the morphologies present and the use of a non-homologous semi-landmark model, these authors describe the differences between the two BSMs samples in order to prove the degree of resolution of their method. Their success rate in accurate classification is 88%. Here, we have reported on 3D and 2D methods that allow a correct classification of >95% of cut and slicing marks. This shows that: (a) 2D sections of marks do capture the essential morphology of the mark without any distortion; (b) the use of semi-landmarks does not create any methodological bias; and (c) no improvement is detected through the use of the complete mark 3D surface.

This shows that no geometric morphometric method is best for classifying cut marks. Taphonomists can dispose of several options depending on their resources. Photogrammetric 2D methods, which require less investment than any of the alternative methods, yield equally accurate (or even slightly better) results than more sophisticated 3D models. Ideally, one should combine methods and select the one producing the best results (i.e., lowest error or highest classification accuracy) in each case, as statisticians do with machine learning methods.

## Additional Information and Declarations

### Data Availability

The following information was supplied regarding data availability: Courtenay, Lloyd (2018): PC Scores. figshare. Dataset. https://doi.org/10.6084/m9.figshare.6146291.v1
Courtenay, Lloyd (2018): 2D Landmarks.txt. figshare. Dataset. https://doi.org/10.6084/m9.figshare.5873838.v1
Courtenay, Lloyd (2018): 3D Landmarks.txt. figshare. Dataset.
https://doi.org/10.6084/m9.figshare.5873832.v1
Courtenay, Lloyd (2018): 45° Measurements of Profile.xlsx. figshare. Dataset. https://doi.org/10.6084/m9.figshare.5873829.v1
Courtenay, Lloyd (2018): 90° Measurements of Profile.xlsx. figshare. Dataset. https://doi.org/10.6084/m9.figshare.5873835.v1.

## References

[ref-1] Aramendi J, Maté-González MÁ, Yravedra J, Cruz Ortega M, Arriaza MC, González-Aguilera D, Baquedano E, Domínguez-Rodrigo M (2017). Discerning carnivore agency through the three-dimensional study of tooth pits: Revisiting crocodile feeding behaviour at FLK-Zinj and FLK NN3 (Olduvai Gorge, Tanzania). Palaeogeogaphy, Palaeoclimatology, Palaeoecology.

[ref-2] Archer W, Braun DR (2013). Investigating the signature of aquatic resource use within pleistocene hominin dietary adaptations. PLOS ONE.

[ref-3] Arriaza MC, Yravedra J, Domínguez-Rodrigo M, Maté-González MA, García-Vargas E, Palomeque JF, Aramendi J, González-Aguilera D, Baquedano E (2017). On applications of micro-photogrammetry and geometric morphometrics to studies of tooth mark morphology: the modern Olduvai Carnivore Site (Tanzania). Palaeogeogaphy, Palaeoclimatology, Palaeoecology.

[ref-4] Bartelink EJ, Wiersema JM, Demaree RS (2001). Quantitative analysis of sharp-force trauma: an application of scanning electron microscopy in forensic anthropology. Journal of Forensic Sciences.

[ref-5] Behrensmeyer AK, Bonnichsen R, Sorg MH (1984). Nonhuman Bone modification in Miocene fossils from Pakistan. Bone Modification.

[ref-6] Behrensmeyer AK, Gordon KD, Yanagi GT (1986). Trampling as a cause of bone surface damage and pseudo-cutmarks. Nature.

[ref-7] Bello SM, Ashton N, Lewis SG, Stringer C (2011). New results from the examination of cut-marks using three-dimensional imaging. The ancient human occupation of Britain.

[ref-8] Bello SM, De Groote I, Delbarre G (2013). Application of 3-dimensional microscopy and micro-CT scanning to the analysis of Magdalenian portable art on bone and antler. Journal of Archaeological Science.

[ref-9] Bello SM, Parfitt SA, Stringer CB (2009). Quantitative micromorphological analyses of cut marks produced by ancient and modern handaxes. Journal of Archaeological Science.

[ref-10] Bello SM, Soligo C (2008). A new method for the quantitative analysis of cutmark micromorphology. Journal of Archaeological Science.

[ref-11] Binford LR (1981). Bones: ancient men, modern myths.

[ref-12] Bonney H (2014). An investigation of the use of discriminant analysis for the classification of blade edge type from cut marks made by metal and bamboo blades. American Journal of Physical Anthropology.

[ref-13] Bookstein F (1989). Principal warps: thin-plate spline and the decomposition of deformations. Transactions on Pattern Analysis and Machine Intelligence.

[ref-14] Boschin F, Crezzini J (2012). Morphometrical analysis on cut marks using a 3D digitalmicroscope. International Journal of Osteoarchaeology.

[ref-15] Capaldo SD (1998). Simulating the formation of dual-patterned archaeofaunal assemblages with experimental control samples. Journal of Archaeological Science.

[ref-16] Chartier S, Allarie JF (2007). Power estimation in multivariate analysis of variance. Tutorials in Quantitatives Methods for Psychology.

[ref-17] Choi K, Driwantoro D (2007). Shell tool use by early members of Homo erectus in Sangiran, central Java, Indonesia: cut mark evidence. Journal of Archaeological Science.

[ref-18] Cohen J (1988). Statistical power analysis for behavioural sciences.

[ref-19] Courtenay LA, Yravedra J, Mate-Gonzalez MA, Aramendi J, González-Aguilera D (2017). 3D analysis of cut marks using a new geometric morphometric methodological approach. Journal of Archaeological Anthropological Sciences.

[ref-20] Crezzini J, Boschin F, Wierer U, Boscato P (2014). Wild cats and cut marks: exploitation of *Felis silvestris* in the Mesolithic of Galgenbühel /Dos de la Forca (Tirol del Sur, Italia). Quaternary International.

[ref-21] De Juana S, Galán AB, Domínguez-Rodrigo M (2010). Taphonomic identification of cut marks made with lithic handaxes: an experimental study. Journal of Archaeological Science.

[ref-22] Domínguez-Rodrigo M (1997). Meat eating by early hominids at FLK Zinj 22 Site, Olduvay Gorge Tanzania: an experimental a roach using cut-mark data. Journal of Human Evolution.

[ref-23] Domínguez Rodrigo M (2002). Hunting and scavenging by early humans: the state of debate. Journal of World Archaeology.

[ref-24] Domínguez-Rodrigo M, Barba R (2005). A study of cut marks on small-sized carcasses and its application to the study of cut-marked bones from small mammals at the FLK Zinj site. Journal of Taphonomy.

[ref-25] Domínguez-Rodrigo M, De Juana S, Galán AB, Rodríguez M (2009). A new protocol to differentiate trampling marks from butchery cut marks. Journal of Archaeological Science.

[ref-26] Domínguez-Rodrigo M, Yravedra J (2009). Why are cut mark frequencies in archaeofaunal assemblages so variable? A multivariate analysis. Journal of Archaeological Science.

[ref-27] Dryden IL, Mardia KV (1998). Statistical shape analysis.

[ref-28] During EM, Nilsson L (1991). Mechanical surface analysis of bone: a case study of cut marks and enamel hypoplasia on a Neolithic cranium from Sweden. American Journal of Physical Anthropology.

[ref-29] Efron B, Stein C (1981). The jackknife estimate of variance. The Annals of Statistics.

[ref-30] Faul F, Erdfelder E, Lang AG, Buchner A G*Power 3: a flexible statistical power analysis program for the social, behavioral, and biomedical sciences. Behavior Research Methods.

[ref-31] Fiorillo AR (1984). An introduction to the identification of trample marks. Current Research.

[ref-32] Fisher DC (1995). Bone surface modifications in zooarchaeology. Journal of Archaeological Method and Theory.

[ref-33] Friedrich S, Konietschke F, Pauly M (2018). Analysis of multivariate data and repeated measures designs with the R package MANOVARM.

[ref-34] Fritz C (1999). Towards the reconstruction of Magdalenian artistic techniues: the ciontribution of microscopic analyses of mobiliary art. Cambridge Archaeological Journal.

[ref-35] Galán AB, Domínguez-Rodrigo M (2013). An experimental study of the anatomical distribution of cut marks created by filleting and disarticulation on the long bone ends. Archaeometry.

[ref-36] Gifford-Gonzalez D, Bonnichsen R, Sorg M (1989). Ethnographic analogues for interpreting modified bones: some cases from East Africa. Bone modification.

[ref-37] Gilbert WH, Richards GD (2000). Digital imaging of bone and tooth modification. The Anatomical Record.

[ref-38] Greenfield HJ (1999). The origins of metallurgy: distinguishing stone from metal cut-marks on bones from archaeological sites. Journal of Archaeological Science.

[ref-39] Greenfield HJ (2006). Slicing cut marks on animal bones: diagnostics for identifying stone tool type and raw material. Journal of Field Archaeology.

[ref-40] Kaiser TM, Katterwe H (2001). The application of 3D-Microprofilometry as a tool in the surface diagnosis of fossil and sub-fossil vertebrate hard tissue. An example from the Pliocene Upper Laetoli Beds, Tanzania. International Journal of Osteoarchaeology.

[ref-41] Klingenberg H (2011). MorphoJ: an integrated software package for geometric morphometrics. Molecular Ecology Resources.

[ref-42] Korkmaz S, Goksuluk D, Zararsiz G (2014). MVN: an R package for assessing multivariate normality. The R Journal.

[ref-43] Lartet E, Heizer RF (1860). On the coexistence of man with certain extinct quadrupeds, proved by fossil bones from various pleistocene deposits, bearing incisions made by sharp instruments. Man discovery of his past.

[ref-44] Lartet E, Christy H (1875). Reliquiae Acquitanicae being contributions to the Archaeology and Paleontology of Perigord and adjoining provinces of Southern France.

[ref-45] Lê S, Josse J, Husson F (2008). FactoMineR: an r package for multivariate analysis. Journal of Statistical Software.

[ref-46] Lewis JE (2008). Identifying sword marks on bone: criteria for distinguishing between cut marks made by different classes of bladed weapons. Journal of Archaeological Science.

[ref-47] Lupo K (1994). Butchering marks and carcass acquisition strategies: distinguishing hunting from scavenging in archaeological contexts. Journal of Archaeological Science.

[ref-48] Lupo KD, O’Connell JF (2002). Cut and tooth mark distributions on large animal bones: ethnoarchaeological data from the Hadza and their implications for current ideas about early human carnivore. Journal of Archaeological Science.

[ref-49] Lyman RL (1987). Archaeofaunas and butchery studies: a taphonomic perspective. Advances in Archaeological Method Theory.

[ref-50] Marín-Monfort MD, Pesquero MD, Fernández-Jalvo Y (2013). Compressive marks from gravel substrate on vertebrate remains: a preliminary experimental study. Quaternary International.

[ref-51] Martin H (1906). Presentation d’ossement de rene pertante des lesions d’origene humaine et animale. Bulletin de la Societé Préhistorique Française.

[ref-52] Martin H, Bourlon MM, Giraux L, Martin H (1907). Presentation d’ossements utilises de l’epoque Musterienne. Un os utilise presolutrean a propos de os utilises.

[ref-53] Martin H (1907-10). Recherches sur l’evolution du Musterien dans le gisement de la Quina (Charente).

[ref-54] Martin H (1909). Desarticulation des quelques regions chez les rumiants et le cheval a l’epoque mousterienne. Bulletin de la Societé Préhistorique Française.

[ref-55] Maté-González MA, Aramendi J, Yravedra J, Blasco R, Rosell J, González-Aguilera D, Domínguez-Rodrigo M (2017b). Assessment of statistical agreement of three techniques for the study of cut marks: 3D digital microscope, laser scanning confocal microscopy and micro-photogrammetry. Journal of Microscopy.

[ref-56] Maté-González MA, Aramendi J, Yravedra J, González-Aguilera D (2017c). Statistical comparison between low-cost methods for 3D characterization of cut-marks on bones. Remote Sensing.

[ref-57] Maté-González MA, Palomeque-González JF, Yravedra J, González-Aguilera D, Domínguez-Rodrigo M (2016). Micro-photogrammetric and morphometric differentiation of cut marks on bones using metal knives, quartzite and flint flakes. Journal of Archaeological and Anthropological Science.

[ref-58] Maté-González MA, Yravedra J, González-Aguilera D, Palomeque-González JF, Domínguez-Rodrigo M (2015). Microphotogrammetric characterization of cut marks on bones. Journal of Archaeological Science.

[ref-59] Maté-González MA, Yravedra J, Martín-Perea D, Palomeque-González J, San-Juan-Blazquez M, Estaca-Gómez V, Uribelarrea D, Álvarez Alonso D, Cuartero F, González-Aguilera D, Domínguez-Rodrigo M (2017a). Flint and quartzite: distinguishing raw material through bone cut marks. Archaeometry.

[ref-60] Nilssen PJ (2000). An actualistic butchery study in South Africa and its implications for reconstructing hominid strategies of carcass acquisition and butchery in the upper Pleistocene and Plio-Pleistocene. PhD dissertation.

[ref-61] O’Higgins P, Jones N (1998). Facial growth in *Cercocebus torquatus*: an application of three dimensional geometric morphometric techniques to the study of morphological variation. Journal of Anatomy.

[ref-62] Olsen SL (1988). The identification of stone and metal tool marks on bone artefacts. Scanning Electron Microscopy in Archaeology.

[ref-63] Olsen SL, Shipman P (1988). Surface modification on bone: trampling Vs butchery. Journal of Archaeological Science.

[ref-64] Otárola-Castillo E, Torquato M, Hawkins HC, James E, Harris J, Marean C, McPhernon S, Thompson J (2018). Differentiating between cutting actions on bone using 3D geometric morphometrics and Bayesian analyses with implications to human evolution. Journal of Archaeological Science.

[ref-65] Pante MC, Muttart MV, Keevil TL, Blumenschine RJ, Njau JK, Merritt SR (2017). A new high-resolution 3-D quantitative method for identifying bone surface modifications with implications for the Early Stone Age archaeological record. Journal of Human Evolution.

[ref-66] Peale J (1870). On the uses of the brain and marrow of animals among the Indians of North America. Smithsonian institution annual report for 1870.

[ref-67] Pineda A, Saladie P, Vergés JM, Huguet R, Cáceres I, Valleverdú J (2014). Trampling versus cut marks on chemically altered surfaces: an experimental approach and archaeological application at the Barranc de la Boella site (la Canonja, Tarragona, Spain). Journal of Archaeological Science.

[ref-68] R Core Team (2014). A language and environment for statistical computing.

[ref-69] Rohlf FJ (1999). Shape statistics: procrustes superimpositions and tangent spaces. Journal of Classification.

[ref-70] Shipman P (1981). Life history of a fossil. An introduction to taphonomy and paleoecology.

[ref-71] Shipman P, Rose J (1983). Early hominid hunting, butchering and carcass-processing behaviours: a roaches to the fossil record. Journal of anthropological Archaeology.

[ref-72] Slice DE (2001). Landmark coordinates aligned by procrustes analysis do not lie in kendall’s shape space. Systematic Biology.

[ref-73] Smith MJ, Brickley MB (2004). Animals and interpretation of flint toolmarks found on bones from West Tump Long Barrow, Gloucestershire. International Journal of Osteoarchaeology.

[ref-74] Walker PL (1978). Butchering and stone tool function. American Antiquity.

[ref-75] Wallduck J, Bello S (2018). Cut mark micro-morphometrics associated with the stage of carcass decay: a pilot study using 3D microscopy. Journal of Archaeological Science: Reports.

[ref-76] West J, Louys J (2007). Differentiating bamboo from stone tool cut marks in the zooarchaeological record, with a discussion on the use of bamboo knives. Journal of Archaeological Science.

[ref-77] White TE (1952). Observations on the butchering technique of some aboriginal peoples, 1. American Antiquity.

[ref-78] White TE (1953). Observations on the butchering technique of some aboriginal peoples, 2. American Antiquity.

[ref-79] White TE (1954). Observations on the butchering technique of some aboriginal peoples, 3, 4, 5, 6. American Antiquity.

[ref-80] White TE (1955). Observations on the butchering technique of some aboriginal peoples, 7, 8, 9. American Antiquity.

[ref-81] Wickham H (2009). ggplot2: elegant graphics for data analysis.

[ref-82] Yravedra J, Diez-Martín F, Egeland CP, Maté-González MA, Palomeque-González JF, Arriaza MC, Aramendi J, Vargas EGarcía, Estaca-Gómez V, Sánchez P, Fraile C, Duque J, Rodríguez SdeFrancisco, González-Aguilera D, Uribelarrea D, Mabulla A, Baquedano E, Domínguez-Rodrigo M (2017b). FLKWest (Lower Bed II, Olduvai Gorge, Tanzania): a newearly Acheulean site with evidence for human exploitation of fauna. BOREAS.

[ref-83] Yravedra J, Maté-González MA, Palomeque JF, Aramendi J, Estaca-Gómez V, San-Juan-Blázquez M, García-Vargas E, Organista E, González-Aguilera D, Arriaza MA, Cobo-Sçanchez L, Gidna A, Uribelarrea D, Baquedano E, Mabulla A, Domínguez-Rodrigo M (2017a). A new approach to raw material use in the exploitation of animal carcasses at BK (Upper Bed II, Olduvai Gorge, Tanzania): a micro-photogrammetric and geometric morphometric analysis of cut marks. BOREAS.

[ref-84] Yravedra J, García Vargas E, Maté-González MA, Aramendi J, Palomeque-González J, Vallés-Iriso J, Matasanz-Vicente J, González-Aguilera D, Domínguez-Rodrigo M (2017c). The use of micro-photogrammetry and geometric morphometrics for identifying carnivore agency in bone assemblage. Journal of Archaeological Science Reports.

